# Positive feedback regulation between USP15 and ERK2 inhibits osteoarthritis progression through TGF-β/SMAD2 signaling

**DOI:** 10.1186/s13075-021-02456-4

**Published:** 2021-03-16

**Authors:** Wenjuan Wang, Yanhui Zhu, Zhenyu Sun, Chen Jin, Xiang Wang

**Affiliations:** 1 Shanghai Key Laboratory of Orthopaedic Implants, Shanghai Ninth People’s Hospital, Shanghai Jiao Tong University School of Medicine, Shanghai, China; 2 Department of Orthopaedic Surgery, Shanghai Ninth People’s Hospital, Shanghai Jiao Tong University School of Medicine, Shanghai, China

**Keywords:** Osteoarthritis, Adeno-associated virus, USP15, ERK2, TGF-β/SMAD2 signaling, Deubiquitination, Positive feedback regulation

## Abstract

**Background:**

The transforming growth factor-β (TGF-β) signaling pathway plays an essential role in maintaining homeostasis in joints affected by osteoarthritis (OA). However, the specific mechanism of non-SMAD and classical SMAD signaling interactions is still unclear, which needs to be further explored.

**Methods:**

In ATDC5 cells, USP15 overexpression and knockout were performed using the transfected lentivirus USP15 and Crispr/Cas9. Western blotting and immunofluorescence staining were used to test p-SMAD2 and cartilage phenotype-related molecular markers. In rat OA models, immunohistochemistry, hematoxylin and eosin (HE)/Safranin-O fast green staining, and histology were used to examine the regulatory activity of USP15 in TGF-β/SMAD2 signaling and the cartilage phenotype. Then, ERK2 overexpression and knockout were performed. The expressions of USP15, p-SMAD2, and the cartilage phenotype were evaluated in vitro and in vivo. To address whether USP15 is required for ERK2 and TGF-β/SMAD2 signaling, we performed rescue experiments in vitro and in vivo. Immunoprecipitation and deubiquitination assays were used to examine whether USP15 could bind to ERK2 and affect the deubiquitination of ERK2. Finally, whether USP15 regulates the level of p-ERK1/2 was evaluated by western blotting, immunofluorescence staining, and immunohistochemistry in vitro and in vivo.

**Results:**

Our results indicated that USP15 stimulated TGF-β/SMAD2 signaling and the cartilage phenotype. Moreover, ERK2 required USP15 to influence TGF-β/SMAD2 signaling for regulating the cartilage phenotype in vivo and in vitro. And USP15 can form a complex with ERK2 to regulate ubiquitination of ERK2. Interestingly, USP15 did not regulate the stability of ERK2 but increased the level of p-ERK1/2 to further enhance the TGF-β/SMAD2 signaling pathway.

**Conclusions:**

Taken together, our study revealed positive feedback regulation between USP15 and ERK2, which played a critical role in TGF-β/SMAD2 signaling to inhibit OA progression. Therefore, this specific mechanism can guide the clinical treatment of OA.

## Background

Osteoarthritis (OA) is the most common chronic arthritis; it is a complex and multifactorial disorder characterized by excessive degradation of articular cartilage, resulting in pain and disability [1, 2]. Disruption to the balance between anabolic and catabolic signaling pathways causes extracellular matrix damage to articular cartilage [3, 4]. Although many signaling mechanisms contribute to protecting cartilage, the stimulating molecular activities and pathways involved in inhibiting the development of OA remain poorly understood.

The transforming growth factor-β (TGF-β) signaling pathway plays an essential role in maintaining tissue homeostasis through anabolic signaling for cartilage growth and repair [5]. TGF-β signaling is initiated by specific type I and II serine/threonine kinase receptors. The specific receptor-regulated (R)-SMAD proteins SMAD2 and SMAD3 form a complex with the co-SMAD SMAD4 and are phosphorylated via the activated TGF-β type I receptor (TbRI). The SMAD complex is then efficiently transferred into the nucleus to regulate the transcription of target genes [6]. Furthermore, the TGF-β signaling pathway can be modified by ubiquitination to regulate downstream molecules [7]. Deubiquitinating enzymes (DUBs) can regulate target proteins by removing polyubiquitin chains. Recently, knowledge of the ability of DUB USP15 to deubiquitinate and stabilize R-SMADs and TbRI has increased our understanding of TGF-β signaling activity in many diseases, such as glioblastoma and systemic sclerosis fibroblasts [8–10]; this knowledge has attracted our attention in terms of applying this to OA experiments.

Extracellular signal-regulated kinase (ERK), a member of the MAPK family, has been shown to be responsible for maintaining matrix anabolism and homeostasis of chondrocytes [11]. As described previously, ERK is part of the non-SMAD pathway of TGF-β signaling and modulates the TGF-β1/SMAD pathway by enhancing SMAD2/3 transcriptional activity [12, 13]. Notably, crosstalk mechanisms between ERK and SMAD pathways have been reported in many biological responses [14–17]. However, the specific associations between ERK and SMAD pathways in OA have not yet been fully elucidated.

In the present study, we reported that there was positive feedback regulation between USP15 and ERK2 in regulating the TGF-β/SMAD2 signaling and maintaining the cartilage phenotype. This process could serve as an important quality control mechanism for preventing cartilage matrix degradation in OA tissues.

## Methods

### Animals

Two-month-old male Norway rats (280–300 g; *n* = 42) were purchased from Shanghai SLAC Laboratory Animal Co. (Shanghai, China). All rats were randomly assigned to two groups: the sham surgery group (*n* = 14) and the OA model group (*n* = 28). All animal studies were conducted according to the guidelines of animal care and were approved by the Animal Care Committee of Shanghai Jiao Tong University.

### Reagents

The following reagents were used: TGF-β1 (10 ng/mL; R&D Systems, USA), MG132 (5 μM; Sigma-Aldrich, USA), Dulbecco’s modified Eagle’s medium (DMEM, Gibco, Thermo Fisher Scientific, USA), cycloheximide (100 μg/mL; Sigma-Aldrich, USA), and PD98059 (10 μM; Sigma-Aldrich, USA).

### In vivo rat OA model

Twenty-four 2-month-old male Norway rats were anesthetized by intraperitoneal injection with 3% pentobarbital sodium until cessation of all sensory reflexes. The right knees of rats were routinely disinfected with povidone iodine before surgery. The right knee joint was exposed by incision from the medial side of the patella, and the medial collateral ligament was removed to open the articular cavity. The rats then underwent anterior cruciate ligament transection in combination with partial medial meniscectomy (ACLT + pMMx) on the right knee as described previously [18, 19]. Finally, the incisions were sutured, and ACLT + pMMx surgeries were induced after complete hemostasis being achieved. In the control group, the sham operations were performed on the model rats. After 8 weeks, OA models were successfully induced in rats that received the ACLT + pMMx surgeries.

### Preparation of AAV vectors

Adeno-associated virus (AAV) serotype 2 vectors are AAV plasmids that can encode target genes and enhance green fluorescent protein (EGFP) cDNA under control of the cytomegalovirus (CMV) enhancer. According to the previously reported method [20], serotype 2 of AAV (AAV-CMV-rUSP15-FLAG-T2A-EGFP and pAAV-U6-rERK2 [shRNA-1]-EGFP) packaged with two plasmids (pAAV-RC and pAAV-Helper) was obtained. The AAV knockdown sequence targeting ERK2 was 5′-CTTCCAACCTCCTGCTGAA-3′.

### AAV intraarticular injections

The two sub-control groups that received the sham surgeries were detailed as follows: (1) seven rats did not receive any injection and (2) seven rats received a 30 μL intraarticular injection of pAAV-U6-rERK2 [shRNA-1]-EGFP (2.5 × 10^10^ vg) in the right knee joint.

The rat OA model groups that received the ACLT + pMMx surgeries were divided into three groups, as follows: (1) fourteen OA model rats did not receive any injection, (2) seven OA model rats received a 30 μL intraarticular injection of pAAV-CMV-rUSP15-FLAG-T2A-EGFP (2.5 × 10^10^ vg) in the right knee joint, and (3) seven OA model rats received a 30-μL injection of pAAV-CMV-rUSP15-FLAG-T2A-EGFP (2.5 × 10^10^ vg) followed by another 30 μL injection of pAAV-U6-rERK2 [shRNA-1]-EGFP (2.5 × 10^10^ vg).

### Immunohistochemistry, hematoxylin and eosin (HE)/Safranin-O fast green staining, and histology

Knee joints were fixed in 10% neutral buffered formalin for 3 d and decalcified in 10% EDTA solution at 4 °C for 3 months. Samples were dehydrated in ethanol, embedded in paraffin, and cut into coronal sections. Immunohistochemical results were detected with the following antibodies: Aggrecan (ab3778, Abcam), Col2a1 (ab34712, Abcam), MMP13 (ab219620, Abcam), USP15 (ab71713, Abcam), ERK2 (sc-1647, Santa Cruz Biotechnology), p-SMAD2 (ab188334, Abcam), and p-ERK1/2 (#4370S, Cell Signaling Technology). Cartilage was stained with HE/Safranin-O fast green staining to observe general morphological changes. And then hyaline cartilage (HC) thickness was counted by at least three participants. The histological properties of the cartilage were assessed using the Osteoarthritis Research Society International (OARSI) scoring system as previously described [21, 22]. Double-blind histological evaluations of the cartilage lesions were performed during the entire analysis process.

### Cell culture, transient transfections, and lentiviral infections

Primary chondrocytes were isolated from rat cartilage tissue as previously described [23]. Briefly, rats were euthanized by cervical dislocation, and the hyaline cartilage of the femoral and tibial surfaces was removed with a scalpel. The pared hyaline cartilage was digested after cutting into pieces with hyaluronidase and collagenase II treatment. The isolated cells were cultured in DMEM containing 10% fetal bovine serum (FBS) at 37 °C under a humidified atmosphere of 5% CO_2_. To amplify the cultured cells, they were subcultured using 0.25% trypsin/EDTA. The isolated chondrocytes were identified by immunostaining for collagen II and Aggrecan.

ATDC5 cells (Riken Cell Bank, Japan) were obtained and maintained in DMEM/F12 (1:1) (GIBCO, USA) containing 5% FBS (HyClone, GE Healthcare Life Sciences, USA), 100 U/mL penicillin, and 100 μg/mL streptomycin in a humidified incubator with 5% CO_2_ at 37 °C. To induce chondrogenic differentiation, the medium was supplemented with 10 μg/mL of insulin-transferrin-selenium (ITS) (Thermo Fisher Scientific, USA) after confluence, and cells were cultured for 14 days. 293T cells (Riken Cell Bank, Japan) were cultured in high glucose DMEM supplemented with 10% FBS and D-glutamate.

Transient transfections and lentiviral infections were performed as previously described [24]. 293T cells were transiently transfected with pLenti-CMV-mUSP15-FLAG-GFP-Puro, pLenti-CMV-mUSP15C269S-FLAG-GFP-Puro, and pLenti-CMV-mERK2-HA-Puro. PLenti-CMV-mUSP15-FLAG-GFP-Puro, pLenti-CMV-mUSP15C269S-FLAG-GFP-Puro, pLenti-CMV-mERK2-HA-Puro, lentiCRISPRv2-USP15a, lentiCRISPRv2-USP15b, lentiCRISPRv2-ERK2a, lentiCRISPRv2-ERK2b, and pLenti-CMV-Ub-His-Puro were co-transfected into HEK293T cells with the packaging plasmids pVSVg (AddGene 8454) and psPAX2 (AddGene 12260). The relevant CRISPR sequence were as follows: USP15-a-F: 5′-CACCG GGTATCTAGTAGATAGTCGG-3′, USP15-a-R: 5′-AAAC CCGACTATCTACTAGATACCC-3′, USP15-b-F: 5′-CACCG TGGCGACGCGCAGTCACTTA-3′, USP15-b-R: 5′-AAAC TAAGTGACTGCGCGTCGCCAC-3′, ERK2-a-F: 5′-CACCG CAGAGTACGTAGCCACACGT-3′, ERK2-a-R: 5′-AAAC ACGTGTGGCTACGTACTCTGC-3′, ERK2-b-F: 5′-CACCG GGATATACTTTAGCCCTCTC-3′, ERK2-b-R: 5′-AAAC GAGAGGGCTAAAGTATATCCC-3′. ATDC5 cells were infected with the resulting stable lentiviral vectors.

### Quantitative real-time reverse transcription polymerase chain reaction (RT-PCR)

Total RNA was isolated from the ATDC5 cells or articular cartilage in rats using Trizol (TaKaRa, Japan) according to the manufacturer’s instructions. RNA was reverse-transcribed to cDNA using a Reverse Transcription Kit (TaKaRa, Japan). Relative mRNA levels were quantified using a Light Cycler 480 system (Roche, Switzerland) with a SYBR Green I Kit (TaKaRa, Japan). The mouse primer sequences of Col2a1, Col10a1, Sox9, Runx2 and GAPDH used were as follows: Col2a1: 5′-TACTGGAGTGACTGGTCCTAAG-3′ (Forward) and 5′-AACACCTTTGGGACCATCTTTT-3′ (Reverse), Col10a1: 5′-GAATTTCTGTGCCAGGAAAACC-3′ (Forward) and 5′-TTTTCACCTCTTCTTCCCACTC-3′ (Reverse), Sox9: 5′-GAGTTTGACCAATACTTGCCAC-3′ (Forward) and 5′-GTAACTGCCAGTGTAGGTGAC-3′ (Reverse), Runx2: 5′-CCTTCAAGGTTGTAGCCCTC-3′ (Forward) and 5′-GGAGTAGTTCTCATCATTCCCG-3′ (Reverse), and GAPDH: 5′-CACTCTTCCACCTTCGATGC-3′ (Forward) and 5′-TCTTGCTCAGTGTCCTTGCT-3′ (Reverse). The rat primer sequences of Col2a1, Aggrecan, Sox9, and GAPDH used were as follows: Col2a1: 5′-GGAGCAGCAAGAGCAAGGAGAAG-3′ (Forward) and 5′-GGAGCCCTCAGTGGACAGTAGAC-3′ (Reverse), Aggrecan: 5′-GCTACGACGCCATCTGCTACAC-3′ (Forward) and 5′- ATGTCCTCTTCACCACCCACTCC-3′ (Reverse), Sox9: 5′-TGGCAGAGGGTGGCAGACAG-3′ (Forward) and 5′-CGTTGGGCGGCAGGTATTGG-3′ (Reverse), and GAPDH: 5′- ATGGTGAAGGTCGGTGTGAA-3′ (Forward) and 5′-CACCACCCTGTTGCTGTAGC-3′ (Reverse). Relative amounts of mRNA were standardized and calculated as previously described [25, 26].

### Western blotting

Cell extracts were subjected to SDS-PAGE in 10% polyacrylamide gels, followed by blotting onto polyvinylidene difluoride membranes (Millipore, Bedford, USA). The following primary antibodies were used: p-ERK1/2 (#4370S, Cell Signaling Technology), FLAG (#14793S, Cell Signaling Technology), Ubiquitination (#3933, Cell Signaling Technology), GAPDH (#5174, Cell Signaling Technology), ERK2 (sc-1647, Santa Cruz Biotechnology), HA (sc-1647, Santa Cruz Biotechnology), His (sc-8036, Santa Cruz Biotechnology), USP15 (ab71713, Abcam), p-SMAD2 (ab188334, Abcam), SMAD2 (ab40855, Abcam), and SMAD4 (ab40759, Abcam). Corresponding species-specific secondary antibodies (anti-rabbit IgG HRP and anti-mouse IgG HRP) were used. GAPDH was used to normalize the results, adjusting for control variations between individual experiments. The results were detected using a Western Chemiluminescent HRP Substrate kit (Millipore, Burlington, USA) and imaged using the FluorChem M system (Protein Simple, San Jose, USA).

### Immunoprecipitation and deubiquitination assay

Monolayer cultured cells were added to ice cold RIPA buffer and centrifuged at 10,000×*g* for 10 min at 4 °C. The supernatant was transferred to a fresh 1.5 mL conical centrifuge tube on ice with 5 μL of indicated antibodies and 20 μL of resuspended volume of Protein A/G PLUS-Agarose (Santa Cruz Biotechnology), and then incubated overnight at 4 °C on a rocker platform. Immunoprecipitates were collected and the supernatant discarded. The pellet was washed four times 1.0 mL RIPA buffer. After the final wash, samples were boiled for 10 min and analyzed by western blotting. To detect ERK2 deubiquitination, transient transfections and lentiviral stable infections were performed before immunoprecipitation.

### Immunofluorescence staining

Immunofluorescence staining was performed as previously described [27, 28]. In brief, cells were fixed with 4% formaldehyde and incubated with USP15, p-ERK1/2, HA, FLAG, Aggrecan, Col2a1, and Col10a1 antibodies. Immunofluorescence staining results were detected with the following antibodies: Col2a1 (ab34712, Abcam), Aggrecan (ab3778, Abcam), Col10a1 (ab49945, Abcam), and other antibodies were described above. The samples were then treated with a secondary antibody. Nuclear DNA was visualized by DAPI staining and viewed under an immunofluorescence confocal microscope (NIKON Eclipse Ti, Japan).

### Statistical analysis

All data were from at least three independent experiments and are presented as means ± standard deviation (SD). The significance of differences between groups was assessed using the *t* tests using SPSS 13.0 statistical software (SPSS, Chicago, IL, USA). Differences with a *P* < 0.05 were considered statistically significant; *P < 0.05, **P < 0.01, and ***P < 0.001.

## Results

### USP15 overexpression can prevent cartilage damage in vivo and in vitro

**Fig. 1:**
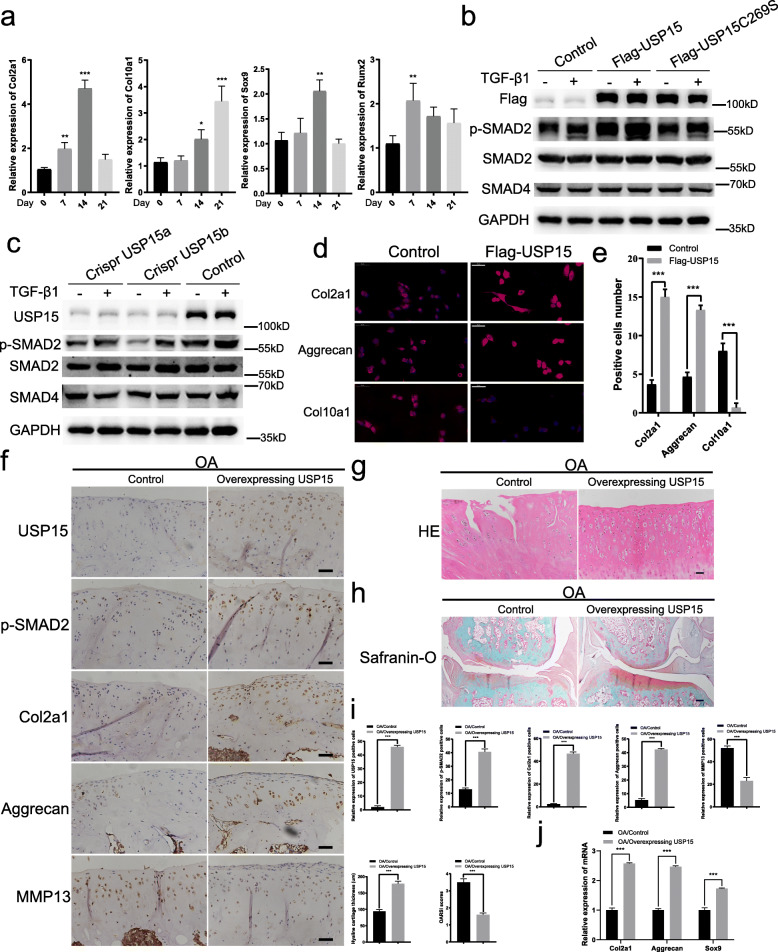
USP15 overexpression can prevent cartilage damage in vivo and in vitro. **a** ITS induction solution was added to induce cartilage phenotype in ATDC5 cells at 7, 14, and 21 days, and quantitative real-time PCR was used to detect the marker molecules related to cartilage phenotype. **b**, **c** Overexpression or knockout of USP15 in ATDC5 cells with TGF-β1 (10 ng/mL). **d** Immunofluorescence staining performed in ATDC5 cells with or without overexpressing USP15 for Col2a1, Aggrecan, and Col10a1 (scale bars = 50 μm). **e** The intensities of immunofluorescence of Col2a1, Aggrecan, and Col10a1 in each group were calculated, and the data were presented as the mean ± SD. **f**–**h** The expression levels of USP15, p-SMAD2, Col2a1, Aggrecan, and MMP13 in the two groups were detected by immunohistochemistry, and the cartilage tissue morphology was detected by HE/Safranin-O fast green staining (*n* = 4 for control groups in the OA models, *n* = 4 for AAV-mediated USP15 overexpression groups in the OA models). **f** Scale bars = 50 μm. **g** Scale bars = 50 μm. **h** Scale bars = 200 μm. **i** The relative expressions of USP15, p-SMAD2, Col2a1, Aggrecan, and MMP13 in each group were calculated via immunohistochemistry, and the hyaline cartilage thickness and OARSI scores were quantified. The data were presented as the mean ± SD. *P < 0.05, **P < 0.01, ***P < 0.001. **j** Relative gene expressions of Col2a1, Aggrecan, and Sox9 associated with cartilage anabolic metabolism in both groups were detected by quantitative real-time PCR (*n* = 3 for control groups in the OA models followed by quantitative real-time PCR, *n* = 3 for AAV-mediated USP15 overexpression groups in the OA models followed by quantitative real-time PCR). The data were presented as the mean ± SD. *P < 0.05, **P < 0.01, ***P < 0.001. All experiments were performed at least three times

We assessed the mechanism by which USP15 regulates the TGF-β signaling pathway. In ADTC5 cells, ITS induction solution was added to continuously induce the cartilage phenotype, and PCR detection of cartilage phenotype-related marker molecules was performed at 7, 14, and 21 days. It was confirmed that the expression levels of Col2a1 and Sox9 peaked at 14 days. Col10a1 peaked after 21 days of induction, and Runx2 decreased gradually after 7 days of induction. According to the results of this experiment and relevant literature reports [29, 30], we set 14 days as the optimal cartilage induction time (Fig. 1a). All the subsequent experiments required ATDC5 cells that had been induced. Overexpression of USP15, rather than USP15 mutant USP15 C269S (enzymatically inactive USP15), led to an increase in phosphorylated SMAD2 (p-SMAD2) (Fig. 1b). In contrast, endogenous USP15 knocked out by Crispr/Cas9 in untreated and TGF-β1-treated ATDC5 cells decreased the level of p-SMAD2 instead of decreasing SMAD2 and SMAD4 expression (Fig. 1c). Compared with the role of Crispr USP15a, Crispr USP15b decreased p-SMAD2 in a more obvious manner; we therefore selected the sequence of Crispr USP15b for subsequent experiments. Immunofluorescence staining in ATDC5 cells demonstrated that USP15 could increase concentrations of Col2a1 and Aggrecan, which are molecules involved in cartilage anabolic mechanism. Furthermore, we found that the level of Col10a1 was decreased (Fig. 1d, e).

To further determine whether USP15 has an inhibitory effect on chondrocyte destruction, we injected rat OA models with AAV-mediated USP15 overexpression in situ. Immunostaining results demonstrated that there were higher levels of USP15, p-SMAD2, Col2a1, and Aggrecan and lower levels of MMP13 in the AAV-mediated USP15 overexpression groups than in the control groups of the OA models (Fig. 1f, i). Histological analysis was performed using the HE/Safranin-O fast green staining (Fig. 1g–i). Compared to the control group, the stained Safranin-O color of the cartilage in the USP15 overexpression group was deeper and the articular plane was repaired (Fig. 1h). The thickness of hyaline cartilage was measured, and OARSI scoring was conducted for each group according to the staining results. It can be concluded that USP15 overexpression can significantly increase the thickness of hyaline cartilage, and its OARSI score is much lower than that of the control group (Fig. 1i). To further verify the role USP15 in cartilage damage, we detected some molecules that promote anabolic processes in cartilage anabolic by quantitative real-time PCR in rat cartilage tissue. The results showed that USP15 overexpression increased the expression of Col2a1, Aggrecan, and Sox9 (Fig. 1j). Thus, these results showed that USP15 could regulate TGF-β/SMAD2 signaling and prevent cartilage damage in vitro and in vivo.

### ERK2 can regulate USP15 and TGF-β signaling to maintain the cartilage phenotype in vivo and in vitro

**Fig. 2:**
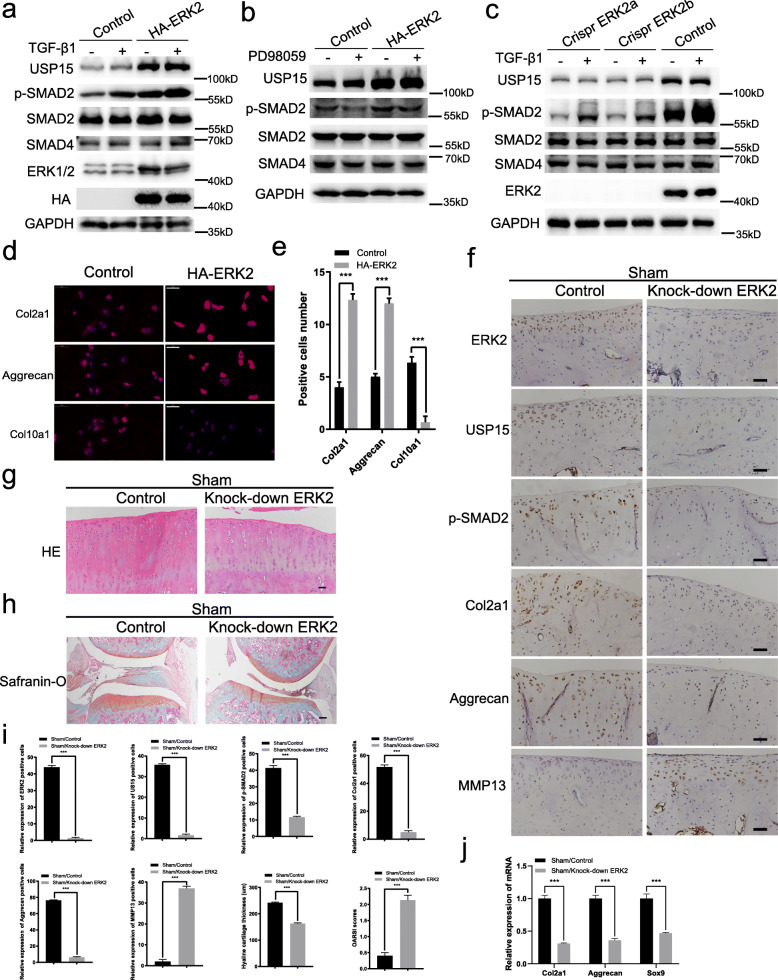
ERK2 can regulate USP15 and TGF-β signaling to maintain the cartilage phenotype in vivo and in vitro. **a**–**c** Overexpression or knockout of ERK2 in ATDC5 cells with TGF-β1 (10 ng/mL) or PD98059 (10 μM). **d** Immunofluorescence staining performed in ATDC5 cells with or without ERK2 overexpression for Col2a1, Aggrecan, and Col10a1 (scale bars = 50 μm). **e** The intensities of immunofluorescence of Col2a1, Aggrecan, and Col10a1 in each group were calculated, and the data were presented as the mean ± SD. **f**–**h** The expression levels of ERK2, USP15, p-SMAD2, Col2a1, Aggrecan, and MMP13 in the two groups were detected by immunohistochemistry, and the cartilage tissue morphology in the two groups were detected by HE/Safranin-O fast green staining (*n* = 4 for control groups in the sham surgery models, *n* = 4 for AAV-mediated ERK2 knockdown groups in the sham surgery models). **f** Scale bars = 50 μm. **g** Scale bars = 50 μm. **h** Scale bars = 200 μm. **i** The relative expressions of ERK2, USP15, p-SMAD2, Col2a1, Aggrecan, and MMP13 in each group were calculated via immunohistochemistry, and the hyaline cartilage thickness and OARSI scores were quantified. The data were presented as the mean ± SD. *P < 0.05, **P < 0.01, ***P < 0.001. **j** Relative gene expressions of Col2a1, Aggrecan, and Sox9, which are associated with cartilage anabolic metabolism, were detected by quantitative real-time PCR in both groups (*n* = 3 for control groups in the sham surgery models followed by quantitative real-time PCR, *n* = 3 for AAV-mediated ERK2 knockdown groups in the sham surgery models followed by quantitative real-time PCR). The data were presented as the mean ± SD. *P < 0.05, **P < 0.01, ***P < 0.001. All experiments were performed at least three times

To determine whether ERK2 can regulate USP15 to influence the TGF-β signaling pathway, we transfected lentivirals to overexpress and knock out ERK2 in ATDC5 cells. When ERK2 was overexpressed, the expression of USP15 was increased in untreated and TGF-β1-treated ATDC5 cells. Subsequently, p-SMAD2 was evidently activated in the TGF-β signaling pathway (Fig. 2a). These results also indicated that the levels of USP15 and p-SMAD2 were suppressed by ERK2 knockout (Fig. 2c). When PD98059 inhibited ERK1/2 phosphorylation, the level of p-SMAD2 was decreased accordingly (Fig. 2b). Furthermore, the total expression of SMAD2 and SMAD4 was unchanged after undergoing these treatments (Fig. 2a–c). Finally, immunofluorescence staining of ATDC5 cell revealed that ERK2 can also maintain the cartilage phenotype and inhibit the destruction factor of cartilage formation, as confirmed by statistical analysis (Fig. 2d, e).

Notably, it was reported that ERK2 is crucial for the activity of R-SMADs [31]. We also observed that AAV-mediated ERK2 knockdown reduced the levels of USP15, p-SMAD2, Col2a1, and Aggrecan and raised the level of MMP13 in rat sham surgery model knee joints (Fig. 2f). Under the influence of ERK2 knockdown, the thickness of the sham surgery cartilage was significantly reduced and the articular surface became rough, as observed via HE/Safranin-O staining (Fig. 2g–i). In the sham surgery models, we performed real-time PCR in the control groups and the AAV-mediated ERK2 knockdown groups of rat cartilage to detect some of the molecules that promote anabolic metabolism of cartilage. The results showed that ERK2 knockdown reduced the expression of Col2a1, Aggrecan, and Sox9 (Fig. 2j). Using in vitro and in vivo experiments, our results showed that ERK2 could regulate the expression of USP15 and TGF-β/SMAD2 signaling and maintain the cartilage phenotype.

### ERK2 requires USP15 to influence the TGF-β signaling for regulating the cartilage phenotype in vivo and in vitro

**Fig. 3:**
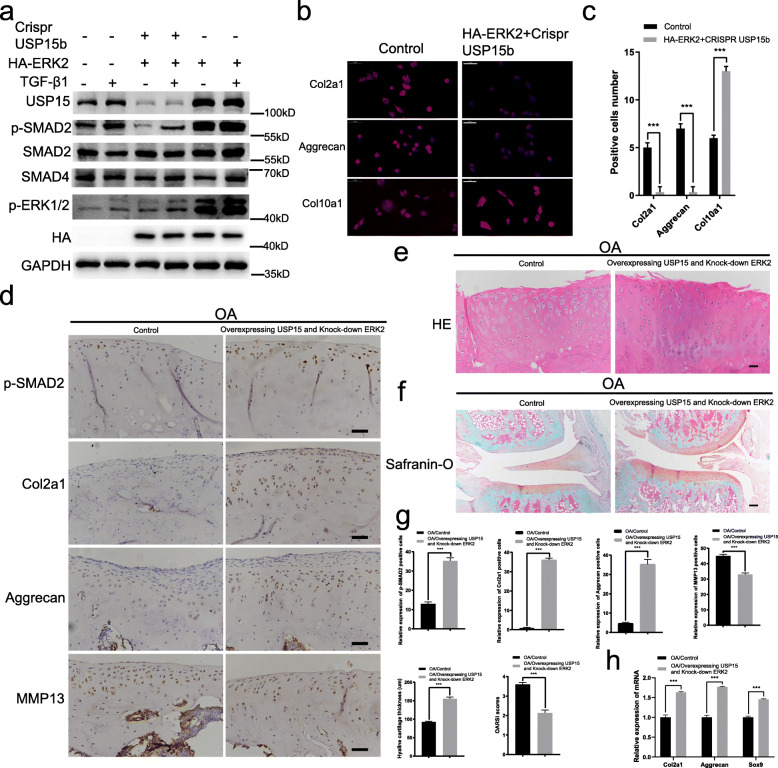
ERK2 requires USP15 to influence the TGF-β signaling for regulating the cartilage phenotype in vivo and in vitro. **a** Simultaneous ERK2 overexpression and USP15 knockout in ATDC5 cells with TGF-β1 (10 ng/mL). **b** Immunofluorescence staining for Col2a1, Aggrecan, and Col10a1 in ATDC5 cells with or without ERK2 overexpression (scale bars = 50 μm). **c** The immunofluorescence intensities of Col2a1, Aggrecan, and Col10a1 in each group were calculated, and the data were presented as the mean ± SD. *P < 0.05, **P < 0.01, ***P < 0.001. **d**–**f** The expression levels of p-SMAD2, Col2a1, Aggrecan, and MMP13 in the two groups were detected by immunohistochemistry, and the cartilage tissue morphology in the two groups were detected by HE/Safranin-O fast green staining (*n* = 4 for control groups in the OA models, *n* = 4 for AAV-mediated USP15 overexpression and ERK2 knockdown groups in the OA models). **d** Scale bars = 50 μm. **e** Scale bars = 50 μm. **f** Scale bars = 200 μm. **g** The relative expressions of p-SMAD2, Col2a1, Aggrecan, and MMP13 in each group were calculated via immunohistochemistry, and the hyaline cartilage thickness and OARSI scores were quantified. The data were presented as the mean ± SD. *P < 0.05, **P < 0.01, ***P < 0.001. **h** Relative gene expressions of Col2a1, Aggrecan, and Sox9, which are associated with cartilage anabolic metabolism, were detected by quantitative real-time PCR in two groups (*n* = 3 for control groups in the OA models followed by real-time quantitative PCR, *n* = 3 for AAV-mediated USP15 overexpression and ERK2 knockdown groups in the OA models followed by real-time quantitative PCR). The data were presented as the mean ± SD. *P < 0.05, **P < 0.01, ***P < 0.001. All experiments were performed at least three times

We transfected lentivirus with ERK2 overexpression and USP15 knockout at the same time in ATDC5 cells. The level of p-SMAD2 declined with decreasing USP15, despite ERK2 overexpression (Fig. 3a). Furthermore, the results showed that the level of p-SMAD2 was increased by the elevation of p-ERK1/2 treated with TGF-β1. Immunofluorescence staining results further revealed that compared to ERK2, USP15 had more influence on maintaining the cartilage phenotype, such as increasing the expression of Col2a1 and Aggrecan; however, this was not the case for Col10a1 expression (Fig. 3b, c). Overall, these results confirmed that USP15 is required for ERK2 to influence the TGF-β/SMAD2 signaling for regulating the cartilage phenotype.

**Fig. 4:**
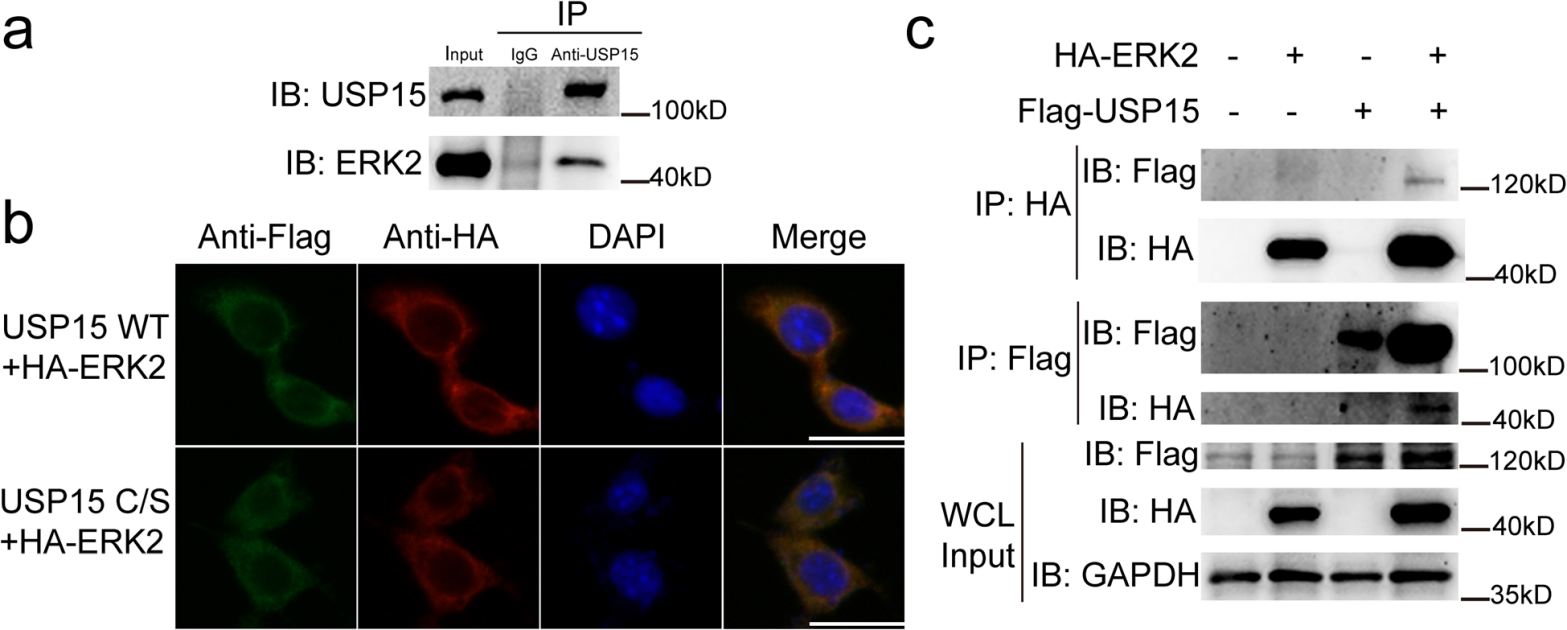
USP15 can form a complex with ERK2. **a** Rat articular chondrocytes were co-immunoprecipitated and examined with the indicated antibodies. Co-immunoprecipitation with anti-USP15 antibodies and immunoblotting with anti-USP15 or ERK2 antibodies. IgG immunoprecipitation was a negative control. **b** Immunofluorescent detection of Flag-USP15, Flag-USP15C269S, and HA-ERK2 in ATDC5 cells. Nuclei was counterstained by DAPI (scale bars = 50 μm). **c** 293T cells were co-transfected with the indicated plasmids, and cell lysates were co-immunoprecipitated with the indicated antibodies and immunoblotted with anti-Flag or anti-HA antibody

To further evaluate the role of USP15 in the TGF-β signaling pathway, we used injections of AAV-mediated USP15 overexpression and ERK2 knockdown simultaneously in the OA rat articular cavity models. According to the results of immunohistochemistry, when overexpressing USP15 and knocking down ERK2 at the same time, the expression of p-SMAD2, Col2a1, and Aggrecan in the cartilage of the knee joint of rats was increased relative to that of MMP13; these results suggest that the TGF-β signaling pathway was strengthened (Fig. 3d). In this rescue case, the HE/Safranin-O staining of the cartilage increased, and we observed that the hyaline cartilage increased in thickness, the articular surface was smoother, and chondrocyte loss decreased (Fig. 3e, f). We then performed a quantitative analysis of hyaline cartilage thickness and OARSI score to further confirm our observation (Fig. 3g). The results of quantitative real-time PCR results showed that when USP15 overexpression was combined with ERK2 knockdown, the expression of Col2a1, Aggrecan, and Sox9 was increased (Fig. 4h). In vivo rat experiments, when USP15 overexpression was combined with ERK2 knockdown in the OA model, injury to the knee cartilage was inhibited. In general, in vitro and in vivo experiments showed that USP15 played a vital role in the transduction of TGF-β/SMAD2 signaling by ERK2 to the downstream cartilage phenotype.

### USP15 can form a complex with ERK2

We investigated the underlying relationship between USP15 and ERK2 by co-immunoprecipitation assays. Indeed, endogenous USP15 can form a complex with endogenous ERK2 in rat articular chondrocytes (Fig. 4a and Supplementary Fig. 1a, b). To further observe the localization of USP15 and ERK2, the results of immunofluorescence staining revealed the colocalization of exogenous ERK2 and USP15 or its mutant in ATDC5 cells (Fig. 4b). The binding interaction was further found between ectopically overexpressed Flag-tagged USP15 and HA-tagged ERK2 by conducting co-immunoprecipitation in 293T cells (Fig. 4c and Supplementary Fig. 1c–f).

### USP15 regulates ERK2 ubiquitination and stimulates p-ERK1/2 levels

**Fig. 5:**
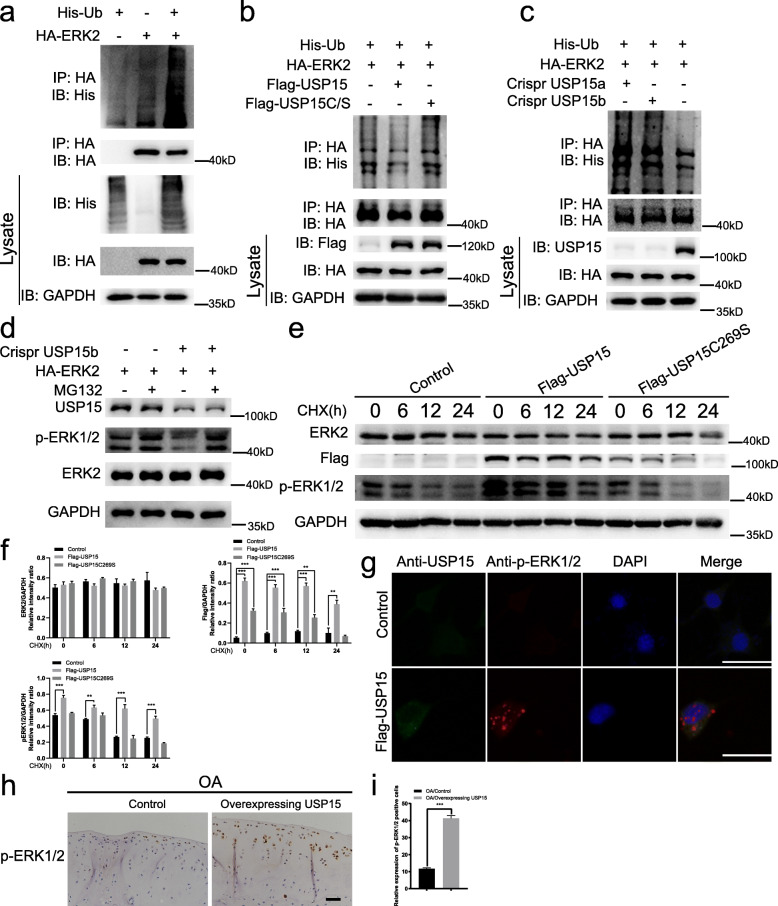
USP15 regulates ERK2 ubiquitination and stimulates p-ERK1/2 levels. **a** ATDC5 cells were infected with stable co-lentiviral vectors HA-ERK2 and His-Ubiquitin (Ub) and immunoprecipitated with HA antibody and Protein A/G PLUS-Agarose, then immunoblotted with antibodies against HA and His. **b** ATDC5 cells were infected with stable co-lentiviral vectors HA-ERK2, His-Ubiquitin (Ub), Flag-USP15, and Flag-USP15C269S. They were immunoprecipitated with HA antibody and Protein A/G PLUS-Agarose, then immunoblotted with antibodies against HA and His. **c** ATDC5 cells were infected with stable co-lentiviral vectors HA-ERK2, His-Ubiquitin (Ub), Crispr USP15a, and Crispr USP15b. They were co-immunoprecipitated with HA antibody and Protein A/G PLUS-Agarose, then immunoblotted with antibodies against HA and His. **d** Immunoblot analysis of ATDC5 cells ERK2 overexpression and Crispr USP15b in the presence or absence of MG132 (5 μM) treatment. **e** ATDC5 cells were infected with stable lentiviral vectors Flag-USP15 and Flag-USP15C269S and treated with cycloheximide (100 μg/mL). Immunoblotting was performed with the indicated antibodies and collected at the indicated times. **f** The quantification data of ERK protein, Flag, and p-ERK1/2 via Immunoblotting. The data were presented as the mean ± SD. *P < 0.05, **P < 0.01, ***P < 0.001. **g** Immunofluorescence and DAPI staining of ATDC5 cells were infected with stable lentiviral vectors Flag-USP15 and then detected by USP15 and p-ERK1/2 antibodies (scale bars = 50 μm). **h** The expression levels of p-ERK1/2 in the two groups were detected by immunohistochemistry (*n* = 4 for control groups in the OA models, *n* = 4 for AAV-mediated USP15 overexpression groups in the OA models) (scale bars = 50 μm). **i** The relative expression of p-ERK1/2 in each group was calculated via immunohistochemistry. The data were presented as the mean ± SD. *P < 0.05, **P < 0.01, ***P < 0.001. Data shown are from one representative experiment out of the three performed

Since USP15 can interact with ERK2, we hypothesized that USP15 deubiquitinates ERK2. First, we co-expressed HA-tagged ERK2 and His-tagged ubiquitination and found that ERK2 could be ubiquitinated in ATDC5 cells (Fig. 5a). Next, HA-tagged ERK2 and His-tagged ubiquitination expression vectors were co-expressed with flag-tagged USP15 wild-type or USP15C269S in ATDC5 cells (Fig. 5b and Supplementary Fig. 1 g, h). Loss of ubiquitination of ERK2 was detected by overexpressing wild-type USP15, but not the catalytically inactive USP15 mutant. Conversely, USP15 depletion markedly enhanced the incorporation of ubiquitin into ERK2 (Fig. 5c and Supplementary Fig. 1i, j).

We therefore investigated whether USP15 affected the stability of ERK2. Surprisingly, the total amount of ERK2 was unchanged by treatment with the proteasome inhibitor MG132 (Fig. 5d). These results indicated that although USP15 can bind to and deubiquitinate ERK2, USP15 was not resistant to degradation by the ERK2 ubiquitination hydrolase. When treated with cycloheximide, overexpressed Flag-tagged USP15 could not protect the half-life of ERK2 (Fig. 5e). However, these data indicated that USP15 increased the stability and half-life of p-ERK1/2 (Fig. 5d–f). Since there is no commercially available p-ERK2 antibody, only the p-ERK1/2 antibody can be used to detect the influence of USP15 on the stability and half-life of p-ERK1/2, which may indirectly reflect the influence of USP15 on these factors. As previously mentioned, USP15 failed to affect ERK2 expression. We evaluated the subcellular distribution of p-ERK1/2 by treatment with overexpressed Flag-tagged USP15 instead of USP15C269S. According to the results of immunofluorescence microscopy, treatment with overexpressed Flag-tagged USP15 increased nuclear p-ERK1/2 levels (Fig. 5g). Furthermore, by means of immunohistochemistry, we verified once again that USP15 overexpression can increase p-ERK1/2 in the OA models compared to in the control groups and the AAV-mediated USP15 overexpression groups (Fig. 5h, i). These results suggest that USP15 regulates ERK2 ubiquitination, but it failed to affect ERK2 degradation via a proteasome. Our results showed that USP15 can affect the p-ERK1/2 level, which may indirectly indicate that USP15 regulates p-ERK2 activation.

## Discussion

OA is the most common type of arthritis with inflammatory disease in the synovial joints [32, 33]. It is characterized by gradual degeneration of articular cartilage and changes in the structure and function of the entire joint [34, 35]. In recent years, it has been reported that canonical SMAD signaling of the TGF-β signaling pathway contributed significantly to cartilage maintenance and repair. To our knowledge, the intricate mechanisms between SMAD and non-SMAD pathways in OA tissues have not yet fully characterized [36–38]. In our study, we further investigated the specific regulatory relationship between USP15 and ERK2 related to the SMAD and non-SMAD pathways to provide an innovative direction for exploring the occurrence and treatment of OA.

USP15 can reportedly act as a responding marker of the TGF-β signaling pathway [39]. Consistent with the in vivo or in vitro measurements, we observed that USP15 stimulated the TGF-β signaling pathway through p-SMAD2 and inhibited OA progression by increasing Col2a1, Aggrecan, and Sox9 levels and suppressing Col10a1 and MMP13 levels. According to these reports, USP15 played indispensable roles in many diseases such as safeguarding genome integrity in leukemia cells, promoting the apoptosis of degenerative nucleus pulposus cells, and enhancing re-epithelialization through deubiquitinating EIF4A1 during cutaneous wound repair [40–43]. However, there are few previous studies on the effects of USP15 on OA. In the present study, we provided evidence supporting the role of USP15 as a new suppressor to ameliorate OA by preventing cartilage damage. ERK2 of non-SMAD pathways is known to regulate the TGF-β signaling pathway through SMADs [44]. Although some diseases such as Marfan syndrome have reported the effect of ERK on the TGF-β signaling pathway [45], we deeply found that ERK2 can affect this pathway through USP15, which is of great significance for supplementing the classical TGF-β signaling pathway. Although ERK2 was overexpressed, the level of p-SMAD2 was decreased due to reduced USP15 expression in rescue experiments. Taken together, these results showed that ERK2 may require USP15 to influence TGF-β/SMAD2 signaling for regulating the cartilage phenotype.

**Fig. 6:**
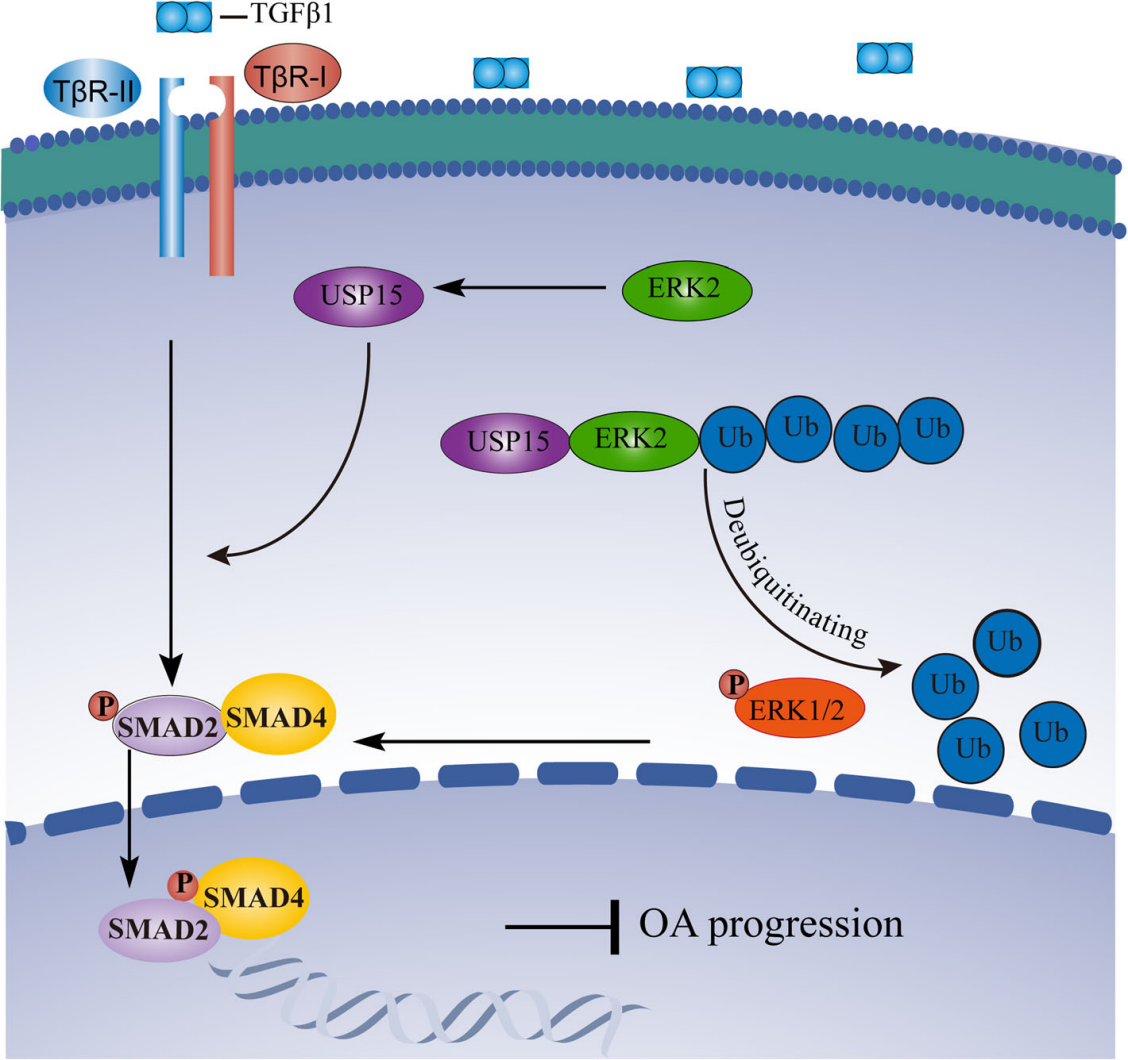
A working model for positive feedback regulation between USP15 and ERK2 playing a critical role in OA. Non-SMAD of ERK2 can promote TGF-β/SMAD2 signaling by increasing the level of USP15. Next, USP15 can interact with deubiquitinate and activate ERK2 to stimulate TGF-β/SMAD2 signaling. Finally, this positive feedback loop can inhibit OA progressions

Co-immunocoprecipitation assays in our current studies showed that USP15 could bind to ERK2. In agreement with our prediction, we analyzed the specific deubiquitination effects of USP15 on ERK2. If USP15 was knocked out, ERK2 underwent more ubiquitination modifications. The most common role in ubiquitination is protein degradation [46–48]. Surprisingly, even though USP15 could deubiquitinate ERK2, the degradation of endogenous ERK2 was not changed by USP15. Since there is no commercially available p-ERK2 antibody, the p-ERK1/2 antibody was used to detect the influence of USP15 on p-ERK1/2 signaling, which may indirectly reflect p-ERK2 signaling. Interestingly, USP15 could regulate the activation of ERK1/2. There is a possibility that ERK2 deubiquitination may promote ERK2 phosphorylation to a certain extent, but the specific role of ubiquitination modification in ERK2 phosphorylation and relevant important modification targets still need to be further explored. According to the previous reports [45, 49, 50] and our results, the activated ERK2 signals lead to enhance p-SMAD2 responses to decrease the cartilage damage. This process results in a positive feedback regulation mechanism between USP15 and ERK2, thus promoting the TGF-β signaling pathway (Fig. 6). Absolutely, more in-depth research combined with clinical studies is required to identify powerful therapeutic targets for OA.

The limitations of this study include the fact that only rat OA models were used to explore deeper mechanisms in further study, and human clinical samples were not included. In addition, primary chondrocytes isolated from rat cartilage tissue were replaced by ATDC5 cells in the part of vitro study because of the difficulty of using chondrocytes for lentiviral infections. Furthermore, there may be some errors in some experimental procedures such as surgical operations of rat models establishment and virus injections, which may affect the biases of the statistical results to a certain extent.

## Conclusions

In conclusion, the present study suggests that positive feedback regulation between USP15 and ERK2 plays a critical role in regulating the TGF-β/SMAD2 signaling and maintaining the cartilage phenotype. Additionally, this cascade of interconnected responses provides new insights into the pathogenesis of OA.

## Supplementary Information

**Additional file 1: Fig. S1.** The full membrane images of immunoprecipitation data for the interaction of USP15 and ERK2. (a–b) Rat articular chondrocytes were co-immunoprecipitated and examined with the indicated antibodies, co-immunoprecipitated with anti-USP15 antibodies, and immunoblotted with anti-USP15 or anti-ERK2 antibodies. IgG immunoprecipitation was a negative control. (c–f) 293T cells were co-transfected with or without HA-ERK2 and Flag-USP15 in four subgroups and co-immunoprecipitated with the indicated antibodies, co-immunoprecipitated with anti-HA or anti-Flag antibodies, and immunoblotted with anti-Flag or anti-HA antibodies. (g–h) ATDC5 cells were infected with stable co-lentiviral vectors HA-ERK2, His-Ubiquitin (Ub), Flag-USP15, and Flag-USP15C269S. They were immunoprecipitated with anti-HA antibody and then immunoblotted with antibodies against HA or His. (i–j) ATDC5 cells were infected with stable co-lentiviral vectors HA-ERK2, His-Ubiquitin (Ub), Crispr USP15a, and Crispr USP15b. They were co-immunoprecipitated with anti-HA antibody and then immunoblotted with antibodies against HA or His.

## Data Availability

The datasets generated and/or analyzed in this study are available from the corresponding author upon reasonable request.

## Abbreviations

TGF-β: Transforming growth factor-β
OA: Osteoarthritis
TbRI: TGF-β type I receptor
DUBs: Deubiquitinating enzymes
ERK: Extracellular signal-regulated kinase
RT-PCR: Reverse transcription polymerase chain reaction
DMEM: Dulbecco’s Modified Eagle Medium
FBS: Fetal bovine serum
ITS: Insulin-transferrin-selenium
ACLT + pMMx: Aterior cruciate ligament transection in combination with partial medial meniscectomy
AAV: Adeno-associated virus
EGFP: Enhance green fluorescent protein
CMV: Cytomegalovirus
HE: Hematoxylin and eosin
HC: Hyaline cartilage
OARSI: Osteoarthritis Research Society International
