# Supplementary figures and images for: Positive feedback regulation between USP15 and ERK2 inhibits osteoarthritis progression through TGF-β/SMAD2 signaling
Source: Arthritis Res Ther. 2021 Mar 16;23:84. doi: 10.1186/s13075-021-02456-4 (PMC7962367; doi:10.1186/s13075-021-02456-4)

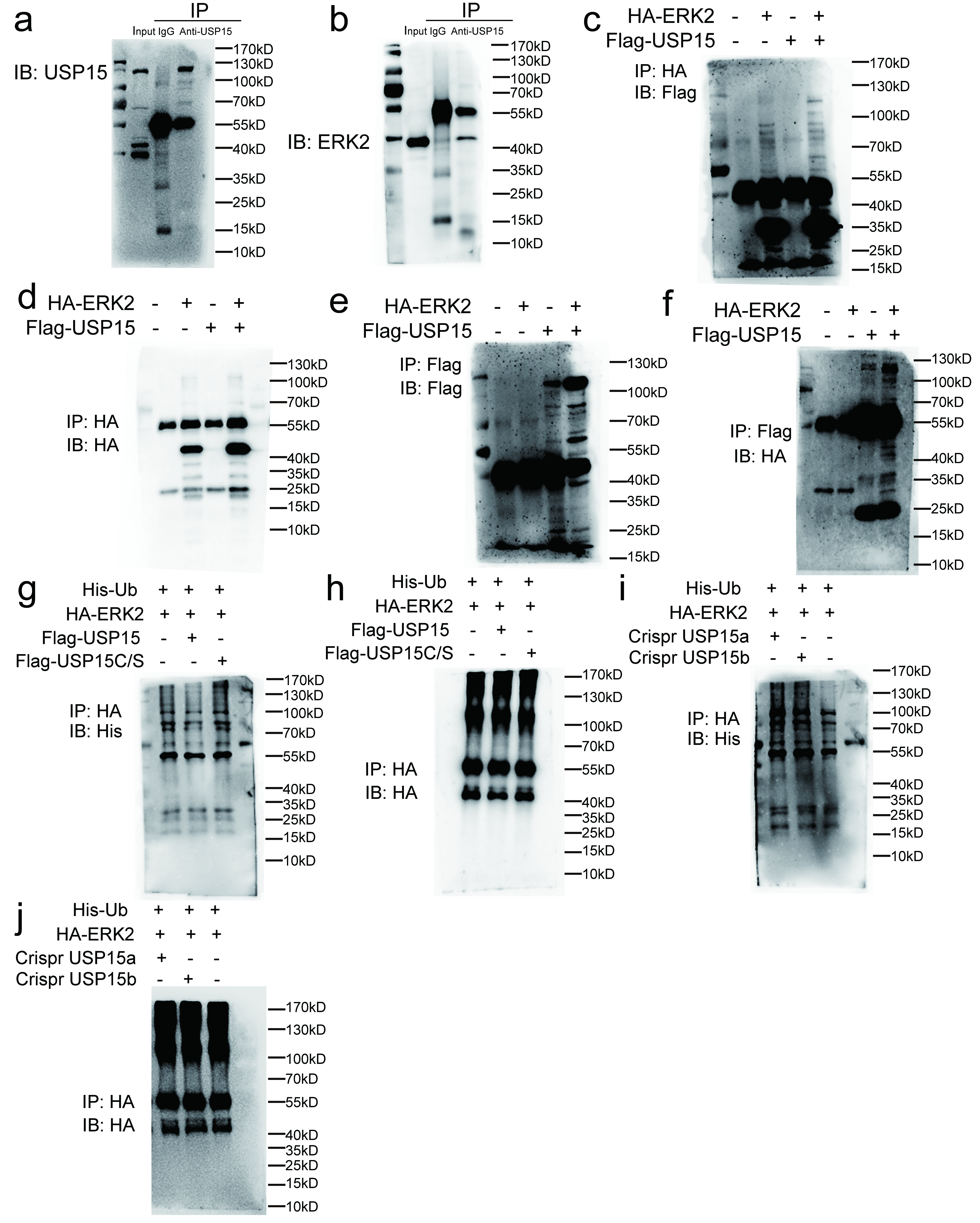

Supplement: Supplementary file 1 — Additional file 1: Fig. S1. The full membrane images of immunoprecipitation data for the interaction of USP15 and ERK2. (a–b) Rat articular chondrocytes were co-immunoprecipitated and examined with the indicated antibodies, co-immunoprecipitated with anti-USP15 antibodies, and immunoblotted with anti-USP15 or anti-ERK2 antibodies. IgG immunoprecipitation was a negative control. (c–f) 293T cells were co-transfected with or without HA-ERK2 and Flag-USP15 in four subgroups and co-immunoprecipitated with the indicated antibodies, co-immunoprecipitated with anti-HA or anti-Flag antibodies, and immunoblotted with anti-Flag or anti-HA antibodies. (g–h) ATDC5 cells were infected with stable co-lentiviral vectors HA-ERK2, His-Ubiquitin (Ub), Flag-USP15, and Flag-USP15C269S. They were immunoprecipitated with anti-HA antibody and then immunoblotted with antibodies against HA or His. (i–j) ATDC5 cells were infected with stable co-lentiviral vectors HA-ERK2, His-Ubiquitin (Ub), Crispr USP15a, and Crispr USP15b. They were co-immunoprecipitated with anti-HA antibody and then immunoblotted with antibodies against HA or His. [file 13075_2021_2456_MOESM1_ESM.tif]
